# Validity and Reliability of Three-chamber-View Three-directional Encoded Phase-contrast Magnetic Resonance Velocity-Vector Mapping for Transmitral Velocity Measurements: Comparison with Doppler Echocardiography and Intra- and Inter-observer Variability

**DOI:** 10.2463/mrms.mp.2015-0172

**Published:** 2016-09-06

**Authors:** Munemura Suzuki, Norihiko Kotooka, Masashi Sakuma, Takahiko Nakazono, Koichi Node, Hiroyuki Irie

**Affiliations:** 1 Department of Radiology, Saga University Hospital, Saga, Japan; 2 Department of Cardiology, Saga University Hospital, Saga, Japan; 3 Suzuki Medical Imaging Lab, 2-8-33 Nishitaniyama, Kagoshima, Kagoshima 891-0117, Japan

**Keywords:** phase-contrast magnetic resonance imaging, three-chamber-view, velocity vector map

## Abstract

**Purpose:**

Three-chamber view (3ch.) three-directional encoded phase-contrast magnetic resonance velocity vector mapping (PCMRVM) has been used for visualization and assessment of intra-cardiac flow. Although transmitral inflow velocity can be determined using this method by tracing mitral tips during the cardiac phase, its feasibility for clinical applications has not been established. Our aim was to investigate the validity and reproducibility of 3ch. PCMRVM for determining transmitral inflow velocity.

**Methods:**

We conducted 3ch. PCMRVM for 32 patients and eight healthy volunteers and analyzed the transmitral inflow pattern and early (E) and late (A) diastolic velocity. Nine patients also underwent Doppler echocardiography to evaluate correlations between the methods for E and A velocities and the E/A ratio. Intra- and inter-observer variability were calculated using intraclass correlation coefficients (ICC [1, 1] and ICC [2, 1]) for peak E and A velocities, Spearman’s rank correlation coefficient for the E/A ratio, and Cohen’s kappa coefficient for the inflow pattern.

**Results:**

Bland-Altman plots indicated that 3ch. PCMRVM showed systemically lower velocities than Doppler echocardiography for E (3 [25.8] 48.6) and A (−6.28 [21] 48.3); however, a strong correlation was observed (*r* = 0.81, *p* < 0.0001). The E/A ratio was not statistically different between the two modalities (*p* = 0.21). The intra- and inter-observer variabilities for peak E and A velocities and the E/A ratio demonstrated nearly perfect agreement or strong correlations, except for the peak E velocity (ICC [2, 1] = 0.751).

**Conclusion:**

Based on these results, 3ch. PCMRVM can be used for both visualization and assessment of intra-cardiac flow and evaluation of the transmitral inflow velocity.

## Introduction

Noninvasive measurement of transmitral flow is indispensable for managing cardiovascular disease and heart failure with normal left ventricular (LV) function.^1^ In clinical practice, Doppler echocardiography is widely used as the standard reference method for measuring transmitral inflow. However, it has some limitations that affect the accuracy of quantification. For example, the quality of the acoustic window and operator experience can affect image quality. In contrast, phase-contrast (PC) magnetic resonance imaging (MRI) can acquire images in any plane or along any selected axis; thus, it can be used as an alternative to Doppler echocardiography.

Conventionally, two-dimensional (2D) one-directional encoded PC MRI of the basal short axial plane is performed and transmitral flow volume is measured to obtain functional parameters.^2^ However, this conventional method has limitations because of the complex multidirectional flow within the heart^2^ and longitudinal cardiac motion.^3–5^ Recently, multislice 2D or 3D three-directional encoded PC MRI has become an important research tool for the cardiovascular system and has provided new insights.^6–9^ Moreover, Westenberg et al. proposed a retrospective valve tracking method using multislice 2D and 3D three-directional encoded PC MRI and demonstrated its effectiveness for evaluating transmitral flow.^3–5^

In this study, we evaluated three-chamber (3ch.) view 2D three-directional encoded PC MR velocity vector mapping (3ch. PCMRVM) for measuring transmitral inflow velocity. As we adapted single slice 2D acquisition for 3ch. PCMRVM, it has short acquisition time compared to multislice 2D and 3D acquisition, and it is possible to trace the mitral tips throughout the cardiac phase. Kim et al.^10^ and Thompson et al.^11^ demonstrated complex flow patterns and pressure differences within the LV using 3ch. PCMRVM; however, its feasibility for clinical application has not been determined. Accordingly, we investigated the reliability and reproducibility of 3ch. PCMRVM. We compared diastolic mitral inflow velocity to that determined by Doppler ultrasound. We also assessed intra- and inter-observer variability for the mitral inflow pattern and velocity.

## Materials and Methods

### Study population

Thirty-two patients (19 men and 13 women, mean age: 59.4 ± 14 years, age range = 17–80) and eight healthy volunteers (five men and three women, mean age = 29.3 ± 5 years, age range = 23–39 years) were included in this study. All patients underwent cardiovascular magnetic resonance (CMR) imaging for various indications, such as investigating the cause of cardiac dysfunction (Table 1). There was no patient who had a valve disease above the moderate grade. 3ch. PCMRVM was performed as part of the clinical study. The study protocol was approved by the Institutional Review Board of Saga University Hospital and written informed consent was obtained from all participants.

### Magnetic resonance image acquisition

All MR images were acquired with a 3 Tesla MR system (MAGNETOM Trio, A Tim System; Siemens AG Healthcare Sector, Erlangen, Germany) using a 32-element phased-array torso-cardiac coil.

3ch. PCMRVM were acquired using 2D segmented fast low-angle shot (2D FLASH) sequences with variable-flow encoding sequences. Velocity was encoded in all three directions (one through-plane and two in-plane directions). In order to avoid the effect of aliasing due to the mismatch between inflow velocity and Velocity encoding (VENC), we determined VENC as follows. First, we set VENC at 150 cm/s. Then we reviewed the acquired image on the console. When we found locally heterogeneous low intensity within the high signal intensity of the main mitral inflow, we regarded it as aliasing. Then, we raised VENC 50 cm/s and repeated the procedure until the low intensity disappeared. The VENC ranged from 150 to 300 cm/s. 3ch. PCMRVM data were acquired during one breath hold. Further imaging parameters were as follows: flip angle = 20°, echo time = 4.28 ms, repetition time = 117.15 ms, field of view = 320–350 mm^2^, slice thickness = 6 mm, matrix = 256 × 256 (phase 68.8%), and k-space segmentation factor = 4, with 20 phases retrospectively reconstructed during one average cardiac cycle (the frame rate is 20 frames/heart beat and a temporal resolution is 117.5 ms for each time frame).

### Doppler echocardiography

Doppler echocardiography was performed by an experienced echocardiographer using Vivid E9 and 7 Dimension systems (GE Healthcare, Milwaukee, WI, USA) with a 2–3.5 MHz transducer. Standard 2D images and Doppler and color-Doppler data were acquired for parasternal and apical views (two-, three-, and four-chamber images were digitally stored in cine-loop format). Transmitral inflow was assessed using the apical three chamber view. The pulsed-wave Doppler was used and 4 mm sample volume was placed at the mitral leaflet tips. The ultrasound Doppler beam was optimized to minimize the intercept angle with mitral flow. The angle correction was not performed. The frame rate of the color Doppler was 19.3 ± 4.2 frames per second. Analyses were subsequently performed offline using EchoPAC v. 108.1.1 (GE Healthcare).

### Phase-contrast magnetic resonance imaging flow analysis

Visualization of 3ch. PCMRVM and measurement of LV diastolic inflow were performed using GTFlow v. 2.2 (GyroTools, Zurich, Switzerland) on a standard personal computer (Intel Core i7 CPU, 3.4 GHz, 16.0 GB RAM).

We did not apply background phase offset error correction techniques for two reasons. First, unlike flow volume measurements, which can have significant errors due to integration over the R-R interval and area of interest, peak velocity measurements may be less subject to small velocity errors caused by phase offset errors.^12^ Second, we measured the inflow velocity over small regions of interest (ROIs) on 2D images near the isocenter of the magnet; 3D acquisition flow analysis with large anatomic coverage tends to have increasing error with greater distance from the isocenter.^13^ The velocity vector field was masked to suppress regions with random phase velocities. The mask was generated by interactively thresholding the corresponding magnitude images at the signal intensity level of the background noise.

**Fig 1.**
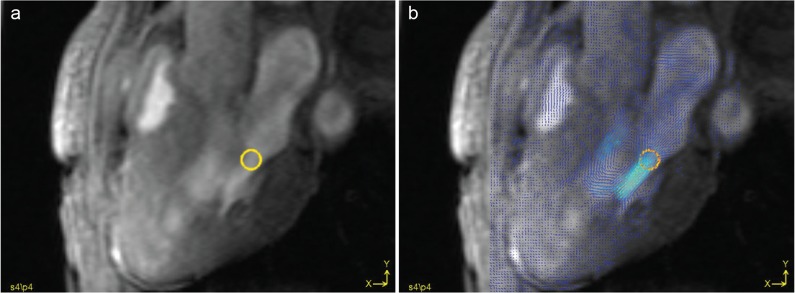
The location of a region of interest at the peak early inflow phase on (**a**) magnitude and (**b**) velocity vector map overlay. A yellow circle between the mitral leaflet tips indicates the region of interest.

After identifying the intra-cardiac anatomy and flow on the image, the observer classified the diastolic inflow pattern as two peaks or a single peak. Then, a round ROI with the same diameter as the opening width of the mitral valve was placed between the mitral tips frame by frame, during each cardiac phase (Fig. 1). The maximum velocity, calculated using the vector sum of the three individual velocity directions within the ROI, was used to create a time–velocity curve. If the diastolic inflow had two peaks, early (E) and late (A) diastolic inflow velocities were calculated along with the E/A ratio; if it had single peak, only the E velocity was calculated.

### Method comparison and intra- and inter-observer variability

To validate the correlation of transmitral inflow parameters between 3ch. PCMRVM and Doppler echocardiography, patients were selected who had undergone both procedures within 7 days and had not changed medications during that time frame in a manner that could modify LV function. Nine patients (six men and three women, mean age = 58.7, age range 36–76) met this criteria. Two observers (M.S. and N.K.) analyzed the 3ch. PCMRVM for these nine patients and eight healthy volunteers by consensus without knowledge of the Doppler echocardiography findings. Thereafter, two observers (M. Suzuki and M. Sakuma) analyzed the 3ch. PCMRVM for all 32 patients independently to investigate inter-observer variability. To calculate intra-observer variability, M.S. repeated the analysis again after a month to avoid any memory effect.

### Statistics

All analyses were conducted using Statcel 3 for Excel 2007 (OMS, Saitama, Japan) and the SPSS statistical package v. 18 (SPSS Inc., Chicago, IL, USA). Continuous variables are expressed as means ± standard deviations. The assumption of normality was tested using the Kolmogorov Smirnov test.

Paired *t*-tests or Wilcoxon signed-rank tests were used to test differences between continuous variables. To determine the relationship between 3ch. PCMRVM and Doppler echocardiography results, Pearson’s correlation coefficients were calculated using a linear regression. Agreement between 3ch. PCMRVM and Doppler echocardiography was also assessed by a Bland-Altman analysis (mean bias in the differences between pairs of measurements) and 95% limits of agreement (±1.96 SD of the difference between pairs of measurements). A *P*-value ≤ 0.05 was used to define statistical significance.

Intra- and inter-observer variability were calculated using intraclass correlation coefficients (ICC [1, 1] and ICC [2, 1]) for peak E and A velocities, Spearman’s rank correlation coefficient for the E/A ratio, and Cohen’s kappa coefficient for the inflow pattern (single peak or two peaks). Correlation was classified as nearly perfect (ICC or kappa = 0.81 – 1.00), substantial (ICC or kappa = 0.61 – 0.80), moderate (ICC or kappa = 0.41 – 0.60), fair (ICC or kappa = 0.21 – 0.40), or slight (ICC or kappa = 0.00 – 0.20).^14^

## Results

**Fig 2.**
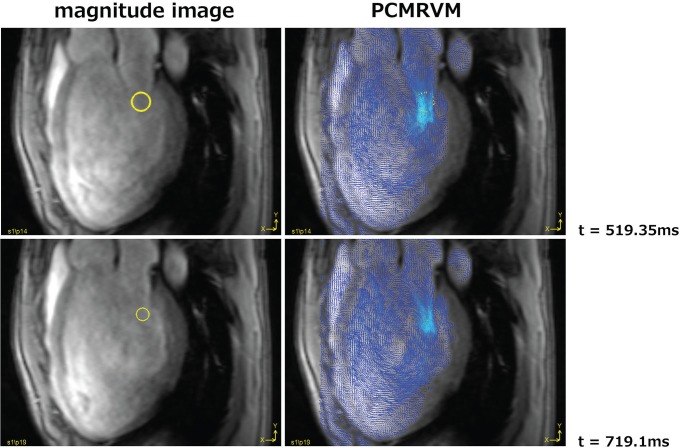
Three-chamber view velocity vector map and time-velocity curve for a patient with DCM. Upper row indicate the magnitude image (left) and PCMRVM at peak early diastolic inflow. Lower row indicate those of late diastolic inflow. DCM, dilated cardiomyopathy; PCMRVM, three-directional encoded phase-contrast magnetic resonance velocity vector mapping. t = 519.35 ms and t = 719.1 ms indicate the time after R wave.

All of the PC MRI examinations were successfully obtained in one breath hold (12.1 – 21.7 sec, average 16.3 sec). The 3ch. PCMRVM were analyzed within 3 min. The peak E and A velocity of eight healthy volunteers were 60.3 ± 13.6 and 27.5 ± 5.4 cm/s. Figure 2 shows magnitude image and 3ch. PCMRVM at the time frame of peak early and late diastolic inflow for a patient with dilated cardiomyopathy (DCM).

### Comparison between three-chamber-view three-directional encoded phase-contrast magnetic resonance velocity vector mapping and Doppler echocardiography

**Fig 3.**
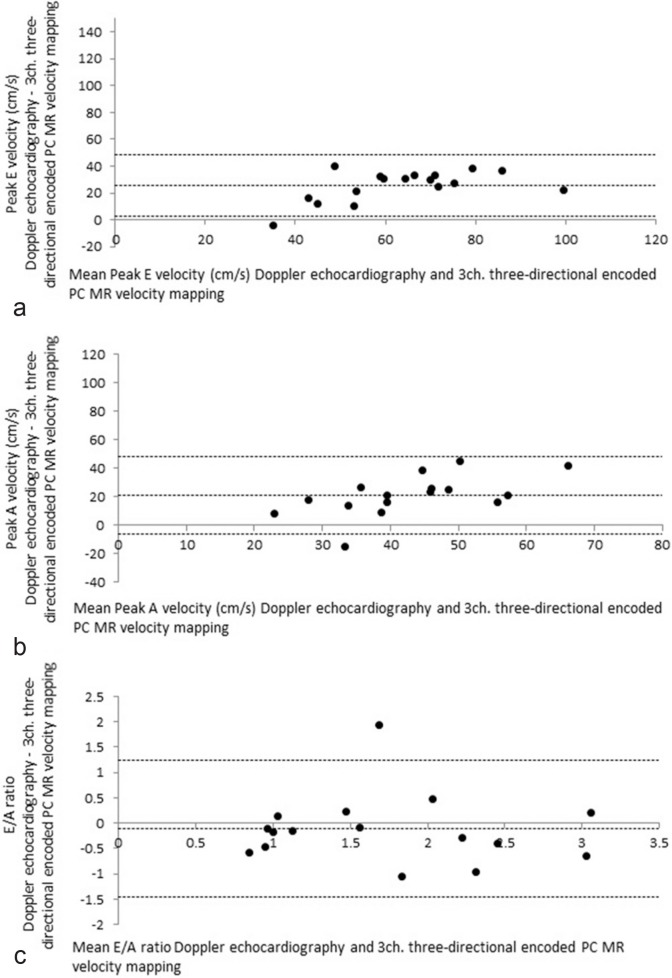
Bland-Altman plots comparing 3ch. PCMRVM and Doppler echocardiography for (**a**) peak E velocity, (**b**) peak A velocity, and (**c**) E/A ratio. 3ch. PCMRVM had a tendency to measure lower peak E and A velocity compared to Doppler echocardiography. However, There was no significant trend for E/A ratio between the two method. The solid line indicates the mean difference and the dashed lines indicate the limits of agreement (±1.96 SD). 3ch, three-chamber view; PCMRVM, three-directional encoded phase-contrast magnetic resonance velocity vector mapping.

**Fig 4.**
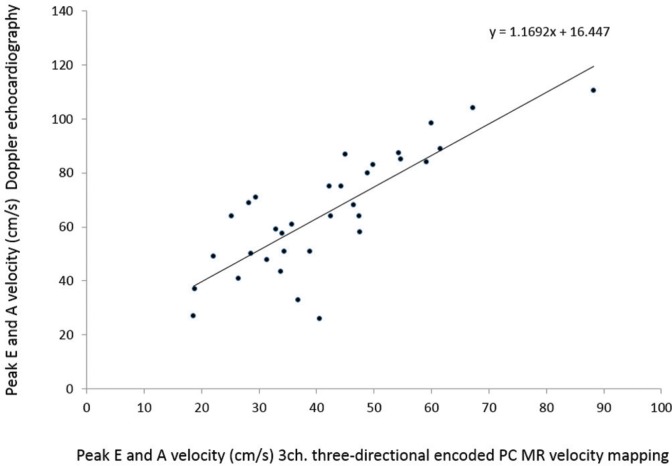
Scatter plot of 3ch. PCMRVM vs. Doppler echocardiography for transmitral inflow velocity. There was a good correlation between the two method for peak E and A velocities. 3ch, three-chamber view; PCMRVM, three-directional encoded phase-contrast magnetic resonance velocity vector mapping.

A single-peak pattern on both 3ch. PCMRVM and Doppler echocardiography was observed in one of the 17 patients. The peak E and A velocities and E/A ratios obtained using both methods for all patients are presented in Table 2. Bland-Altman plots (Fig. 3) indicate that 3ch. PCMRVM shows systemically lower E and A velocities than Doppler echocardiography (E: 3 [25.8] 48.6; A: −6.28 [21] 48.3). However, a scatter plot (Fig. 4) shows a good correlation between the two methods for peak E and A velocities (*r* = 0.81, *p* < 0.001). The E/A ratio was not statistically different between the modalities (*p* = 0.21) and no significant trend was detected based on the Bland-Altman plot (−1.46 [−0.11] 1.24).

### Intra- and inter-observer variability

**Fig 5.**
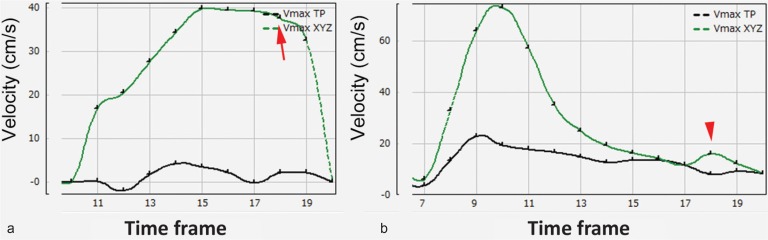
Time-velocity curves for two cases wherein the inflow patterns were classified differently by different observers. (**a**) The small notch during the late diastolic phase. (**b**) The small late diastolic inflow due to the subtle contractile motion of the left atrium. Vmax TP, maximum velocity of through plane direction within the region of interest; Vmax XYZ, maximum velocity of the vector sum of three direction within the region of interest; Time frame corresponds to the reconstructed phase number after the detection of R-wave.

The intra-observer variability showed perfect agreement for classification of the inflow pattern, and the inter-observer agreement was substantial (Tables 3 and 4). The only three cases that were classified differently had a small notch in the time–velocity curve after peak inflow (Fig. 5a, arrow) or a weak late diastolic inflow due to the subtle contractile motion of the left atrium (Fig. 5b, arrowhead).

The peak E and A velocities and E/A ratios for the 32 subjects are summarized in Table 5. Both intra- and inter-observer variability for the peak E and A velocities and E/A ratios demonstrated nearly perfect agreement or very strong correlations, except for the inter-observer variability for the peak E velocity (ICC [2, 1] = 0.751) (Table 6).

## Discussion

The present study demonstrated that 3ch. PCMRVM correlated well with Doppler echocardiography for measuring the E/A ratio. The two methods also had a nearly perfect agreement or very strong correlations for intra-observer variability for the peak E and A velocities, E/A ratio, and flow pattern and for inter-observer variability for the peak A velocity and E/A ratio.

One of our aims was to evaluate the validity of PC MR velocity vector mapping calculated by three-directional encoded PC MRI. Doppler echocardiography and 2D one-directional encoded PC MRI are established methods for evaluating the blood flow. However, as these techniques are one-directional (parallel to the beam or velocity encoded), they are susceptible to mismatch between the acquisition plane and the true direction of flow. Bollache et al. studied transmitral flow using conventional 2D PC MRI and found that a mismatch between the acquisition plane and the true perpendicular to the flow affected the accuracy of the measured velocity compared to the flow rates.^15^ Rathi et al. measured transmitral flow velocity using short axial view three-directional encoded PC MRI and compared the results with those of Doppler echocardiography.^2^ They found excellent agreement for the E/A ratio (bias −0.29), while the peak E and A velocities were lower than those measured by Doppler echocardiography. Although the acquisition plane is different from our study, their results are consistent with the results of our study. Therefore, it suggests that the difference between Doppler ultrasonography and PC MRI is a systemic error.

We discuss the two differences between Doppler and PC MRI. One is the difference of temporal resolution. In order to achieve one-breath hold acquisition, 20 frames were reconstructed for one heartbeat with 117.5 ms of temporal resolution. In comparison with pulsed-wave Doppler echocardiography, both the sampling number and temporal resolution are low. Hence, there is a possibility to fail the maximum velocity of pulsatile flow. Moreover, the velocity measured by retrospective ECG-gated PC MRI is time-averaged velocity of a number of heartbeats. The other is the difference of spatial resolution. Machida et al.^16^ compared PC MRI and intraluminal Doppler guidewire and discussed the importance of high spatial resolution for accurate measurement of pulsatile flow using a small tube phantom. Although the flow profile is different from transmitral inflow, our data might also be affected by the partial volume effect.

The utility of 3ch. was also evaluated. Conventional 3ch. is used in echocardiography and standard CMR (e.g., cine CMR, late gadolinium enhancement) to simultaneously assess the inflow and outflow tract and apex in one imaging plane. Few studies have reported using 3ch. for visualization and assessment of LV flow. Kim et al. investigated the normal diastolic cardiac flow pattern and confirmed a diastolic vortex inside a human LV.^10^ Thomson and McVeigh calculated pressure differences within the cardiac chambers.^11^ However, the clinical feasibility of 3ch. in PC MRI has not been established. In this study, we employed 3ch. to trace the mitral tips visually and placed ROIs in each cardiac phase. Optimization of the mitral valve plane is considered mandatory for accurate transmitral flow measurement when using this method as an alternative to Doppler echocardiography.^3–5^ As we can observe the flow within the left ventricle on 3ch. PCMRVM, it can be used to assess the flow, which is difficult with Doppler echocardiography such as the transmitral inflow affected by the severe aortic regurgitant flow and the apical flow of left ventricular aneurysm with or without thrombus.

Finally, we assessed the advantages and disadvantages of single slice 2D acquisition. Our method requires only a short time for acquisition and analysis and it is not necessary to refer to other images, such as the cine scout view, for identification of anatomy. Thus, a misalignment caused by differences between the image acquisitions is not possible. The primary disadvantage is incomplete spatial coverage. Since the mitral valve area is not completely covered, a transmitral flow rate curve is not available. If the maximum velocity of transmitral flow does not occur within the imaging plane, the E and A velocities will be underestimated.

## Limitations

The present study included a relatively small number of subjects and only a subset of the cases were compared to Doppler echocardiography. Moreover, no patients had valve disease in the study cohort. Assessment of the severity of valve disease is indispensable for the management of cardiovascular diseases such as ischemic heart disease.^17^ To establish the clinical utility of 3ch. PCMRV, further research including evaluation of patients with valve disease is needed.

## Conclusion

We found that 3ch. PCMRVM correlated well with Doppler echocardiography with reference to the E/A ratio. Low inter- and intra-observer variability was also demonstrated. Therefore, this rapid technique can be used for both visualization of intra-cardiac flow and evaluation of transmitral inflow in clinical practice.

## Figures and Tables

**Table 1.** Patient characteristics

Disease	Number (female)	LVEF (%)	LVEDV (ml)	MR none	MR trace	MR mild
DCM	6 (3)	28.5 ± 13.4	211 ± 73.6	3	3	0
HCM	4 (1)	72.3 ± 5.7	90.8 ± 19.1	1	2	1
HHD	6 (0)	27.8 ± 11.1	205.8 ± 20	3	2	1
OMI	4 (1)	53.8 ± 14.5	123.3 ± 57.5	3	1	0
Sarcoidosis	5 (2)	31.2 ± 11.4	93.2 ± 32.1	5	0	0
Others	7 (7)	45.7 ± 22.9	108.4 ± 66	6	1	0

Overall	32 (14)	45.9 ± 21.2	143.2 ± 67.6	21	9	2

DCM, dilated cardiomyopathy; HCM, hypertrophic cardiomyopathy; HHD, hypertensive heart disease; OMI, old myocardial infarction; LVEF, left ventricular ejection fraction; LVEDV, left ventricular end-diastolic volume; MR, mitral regurgitation.

**Table 2.** Peak E and A velocities and the E/A ratio determined by using 3ch. PCMRVM and Doppler echocardiography

	3ch. PCMRVM	Doppler echocardiography	Signed mean diference	*P* value
Peak E velocity (cm/s)	50.5 ± 14.3	76.3 ± 20.3	−25.8	<0.001
Peak A velocity (cm/s)	32.3 ± 9.2	53.3 ± 16.4	−21.0	<0.001
E/A ratio	1.78 ± 0.83	1.66 ± 0.62	−0.11	0.21

**Table 3.** Cross table for classification of the diastolic inflow pattern by one observer

Obs1-1/Obs1-2	Single peak	Two peaks	Total
Single peak	11	0	11
Two peaks	0	21	21
Total	11	21	32

Cohen’s kappa, 1; Obs1-1, first validation of observer 1; Obs1-2, second validation of observer 1.

**Table 4.** Cross table for classification of the diastolic inflow pattern by two observers

Obs1/Obs2	Single peak	Two peaks	Total
Single peak	9	2	11
Two peaks	1	20	21
Total	10	22	32

Cohen’s kappa, 0.788; Obs1, first validation of observer 1; Obs2, validation of observer 2.

**Table 5.** Peak E and A velocities and the E/A ratio measured by two observers

	Obs1-1	Obs1-2	Obs2
Peak E velocity (cm/s)	49.6 ± 11.0	50.2 ± 10.9	49.4 ± 9.56
Peak A velocity (cm/s)	43.8 ± 12.5	43.9 ± 12.8	43.5 ± 11.4
E/A ratio	1.22 ± 0.67	1.28 ± 0.86	1.14 ± 0.49

Obs1-1, first validation of observer 1; Obs1-2, second validation of observer 1; Obs2, validation of observer 2.

**Table 6.** Intra- and inter-observer variability for peak E and A velocities and the E/A ratio

	Peak E velocity ICC (95% CI)	Peak A velocity ICC (95% CI)	E/A ratio (Spearman’s *ρ*)
Intra-observer variability	0.929 (0.86–0.964)	0.976 (0.943–0.990)	0.839
Inter-observer variability	0.751 (0.534–0.876)	0.970 (0.921–0.989)	0.844
